# Long‐term stability of a PTW 34070 large‐area parallel ionization chamber in clinical proton scanning beams

**DOI:** 10.1002/acm2.14525

**Published:** 2024-09-16

**Authors:** Masashi Yamanaka, Yutaro Mori, Kazuki Matsumoto, Shunsuke Moriya, Akihiro Yamano, Takahiro Shimo, Ryosuke Shirata, Kazunori Nitta, Hironori Nagata, Koichi Tokuuye

**Affiliations:** ^1^ Department of Medical Physics Shonan Kamakura General Hospital Kamakura Kanagawa Japan; ^2^ Institute of Medicine University of Tsukuba Tsukuba Ibaraki Japan; ^3^ Department of Radiation Oncology Shonan Kamakura General Hospital Kamakura Kanagawa Japan

**Keywords:** Bragg peak chamber, calibration factor, long‐term stability, proton therapy, response

## Abstract

**Purpose:**

In the modeling of beam data for proton therapy planning systems, absolute dose measurements are performed utilizing a Bragg peak chamber (BPC), which is a parallel‐plate ionization chamber. The long‐term stability of the BPC is crucial for ensuring accurate absolute dose measurement. The study aims to assess the long‐term stability of the BPC in clinical proton pencil beam scanning delivery.

**Methods:**

The long‐term stability evaluation focused on the BPC‐Type 34070 (PTW Freiburg, Germany), utilizing clinical proton scanning beams from December 2022 to November 2023. Monthly investigations were conducted to evaluate the response and cross‐calibration factor of the BPC and a reference chamber, employing the spread‐out Bragg peak (SOBP) field. Additionally, assessments were made regarding the BPC's response to monoenergetic beams, along with an examination of the impact of polarity and ion recombination on the BPC.

**Results:**

The response and cross‐calibration factor of the BPC varied up to 1.9% and 1.8%, respectively, while the response of the reference chamber remained within a 0.5% range. The BPC's response to the mono‐energetic beams varied up to 2.0% across all energies, demonstrating similar variation trends in both the SOBP field and mono‐energetic beams. Furthermore, the variations in polarity and ion recombination effect remained stable within a 0.4% range throughout the year. Notably, the reproducibility of the BPC remained high for each measurement conducted, whether for the SOBP field or mono‐energetic beams, with a maximum deviation observed at 0.1%.

**Conclusions:**

The response and cross‐calibration factor of the BPC demonstrated significant variations, with maximum changes of 1.9% and 1.8%, respectively. However, the reproducibility of the BPC remained consistently high for each measurement. It is recommended that when conducting absolute dose measurements using a BPC, its response should be compared and corrected against the reference chamber for each measurement.

## 1 INTRODUCTION

Calibrating the absolute dose in a treatment planning system (TPS) for proton pencil beam scanning (PBS) delivery requires a dose‐area product to water (DAP_w_) at the reference depth. This DAP_w_ represents the cumulative absorbed dose of water across the plane perpendicular to the proton beam. There exist two primary measurement methods for DAP_w_^1^: (1) Single layer method measures the dose at the center of the two‐dimensional dose distribution.^2^, ^3^ This method involves integrated pencil beams and is utilized to measure DAP_w_ at the center of the field. Farmer or Markus type chambers are commonly employed for this purpose. (2) Single spot method entails the direct measurement of a single pencil beam.^4^, ^5^ In this approach, a large‐area plane‐parallel ionization chamber (LAC), such as a Bragg peak chamber (BPC), is employed to measure the DAP_w_ from a single pencil beam. Calibration of the absorbed dose to water for the LAC is essential for accurate DAP_w_ measurement. Calibration can be achieved through various methods, including cross‐calibration between the LAC and the Farmer chamber, which serves as the reference ionization chamber,^4^, ^6^ or calibration conducted at a dosimetry calibration laboratory by using ^60^Co.^5^ Both of these methods are implemented for absolute dose registration in commercial TPSs.^7^, ^8^, ^9^

Quality assurance (QA) is required to ensure safe and accurate treatments after the beam data is installed in the TPS. The QA program for PBS delivery involves many procedures, including spot position and range. In particular, routine QA of the beam monitor plays an important role because of its direct impact on the accuracy of delivered doses. The accuracy of the beam monitor, a critical aspect of annual QA, is recommended to be within ±2% in the proton PBS delivery method by several guidelines.^10^, ^11^ This stringent tolerance underscores the necessity for precise calibration of the beam monitor. To enable direct comparison of QA results with commissioning and beam data, it is preferable to employ identical measurement method and chamber type. Therefore, ensuring the long‐term stability of both the chamber and treatment machine is crucial for conducting stable QA procedures over an extended duration.

In the single layer method, Farmer and Markus chambers are employed to calibrate the absolute dose of the TPS for PBS proton therapy. The long‐term stability of these chambers has been published by the vendor.^12^ The single spot method, which involves direct measurement of a single beam, employs a BPC, also known as the LAC. However, the long‐term stabilities of certain BPC products remain uncertain, as the original purpose of the BPC is to measure the relative dose distribution, such as the integral depth dose, where long‐term stability is not deemed critical. Nonetheless, the long‐term stability of the BPC cannot be disregarded when utilized in QA procedures to measure the absolute dose in PBS. While there exists a study on the long‐term stability of the BPC using a ^90^Sr check source,^13^ to our knowledge, no study has investigated the long‐term stability of the BPC under the clinical conditions of proton therapy. Furthermore, when conducting absolute dose measurements using the BPC cross‐calibrated with the reference chamber, the long‐term stability of the cross‐calibration factor remains uncertain. Therefore, it is imperative to assess the long‐term stability of the BPC under clinical conditions to accurately measure the absolute dose.

This study aims to evaluate the long‐term stability of the BPC in clinical proton PBS delivery. The long‐term stability of the BPC was evaluated using the response and cross‐calibration factor of the BPC and reference chamber, the response of the BPC to mono‐energetic beams, and the polarity and ion recombination correction factors of the BPC over one year. All verifications were performed at a clinical proton therapy facility.

## 2 MATERIALS AND METHODS

### 2.1 Proton therapy system

Clinical proton beams were measured using a proton therapy treatment machine, specifically the PROBEAT M‐1 (Hitachi, Ltd., Tokyo, Japan), located at Shonan Kamakura General Hospital. This machine has a gantry that can rotates 360°and produces a synchrotron‐based scanning proton beam. The machine always uses a mini‐ridge filter, and a field size is limited to 30 cm × 40 cm at the isocenter. The proton beam energies range from 70.2 to 228.7 MeV. Spot positions are monitored within the nozzle, with a tolerance of these of ±1 mm at the isocenter plane.

### 2.2 Bragg peak chamber

A Bragg peak chamber (Type 34070, PTW Freiburg, Germany) was employed as the LAC. The BPC features a recommended applied voltage of +400 V, an ion collection time of 67 µs, and is suitable for use in both water and air. The BPC has a diameter of 8.16 cm (excluding the guard ring), a cavity height of 2 mm, and a measuring volume of 10.5 cm^3^. Its guard ring is 1 mm wide, while the entrance window of the BPC has a water equivalent thickness (WET) of 4 mm. The BPC was connected to the UNIDOS Tango electrometer (Type 10052, PTW Freiburg, Germany), capable of supplying a variable voltage ranging from −400 to +400 V. Pre‐irradiation procedures involved applying +400 V to the BPC and waiting 10 min before initiating irradiation. The pre‐irradiation conditions were consistent with those detailed for the SOBP field in Section 2.3.1. The BPC was centrally placed within the SOBP and subjected to three irradiation sessions.

### 2.3 Evaluation of long‐term stability

**FIGURE 1:**
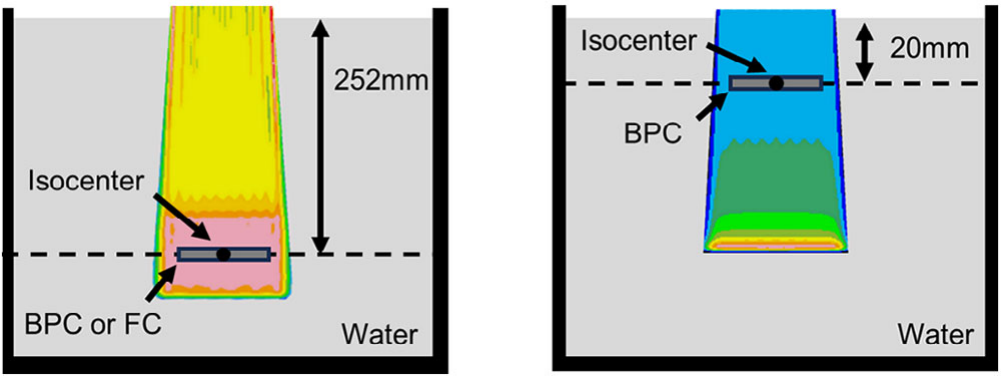
Overview of setup configurations. The left illustrates the setup of the response and cross‐calibration factor between the BPC and reference chamber, Farmer ionization chamber (FC), and *k*_pol_ and *k_s_*, to a SOBP field. The right illustrates the setup of the response of the BPC to mono‐energetic beams.

The long‐term stability of the BPC was assessed using the clinical proton beam.^14^, ^15^ Considering that the response of the chamber could be influenced by factors such as beam quality, dose rate, polarity effect, and ion recombination effects,^6^ the following measurements were performed; (a) response and cross‐calibration factor between the BPC and reference chamber, (b) response of the BPC to mono‐energetic beams, and (c) polarity and ion recombination correction factors (*k*_pol_ and *k_s_*) of the BPC. These measurements were performed monthly over the course of one year, from December 2022 to November 2023, with all measurements conducted at a gantry angle of 0°. All measurements were conducted three times each month. The BPC was positioned on the isocenter plane in a MP3‐PL water tank (PTW Freiburg, Germany), as depicted in Figure 1.

#### 2.3.1 Cross‐calibration of BPC and reference chamber

The response and cross‐calibration factor of the BPC and reference chamber were evaluated over a year (left side of Figure 1). The reference chamber was a farmer ionization chamber (FC) (Type 30013, PTW Freiburg, Germany), featuring a measuring volume of 0.6 cm^3^, a diameter of 3.05 mm, and a length of 23.0 mm. As per technical specifications, the long‐term stability of the FC is reported as 0.5%. The FC is commonly employed for absolute dose measurements. The absorbed dose to water calibration factor of the FC was calibrated by a dosimetry calibration laboratory in Japan. Delivery beams were created using a treatment planning system, VQA Plan (Hitachi, Ltd., Tokyo, Japan). The delivery beam^4^ was spread‐out Bragg peak (SOBP) field optimized using a single field uniform dose with a field size of 11 × 11 × 6 cm^3^. The SOBP dose was 1.0 GyE, with a WET at the SOBP center of 252 mm. Optimization involved utilizing a beam‐specific planning target volume(bsPTV)with a beam lateral margin (spot position error of 2 mm and setup error of the chambers of 1 mm) as well as distal and proximal margins (3.5% of the beam range + 1 mm). The beam energies ranged from 182.7 to 218.7 MeV, with a range shifter of 1 mm. The measurement depth was the center of the SOBP to reduce setup errors and range uncertainty. The calibration factor $N_{D,W,Q_{\mathrm{cross}}}^{\mathrm{BPC}}$ for the proton cross‐calibration quality $Q_{\mathrm{cross}}$ was calculated using the BPC and FC as follows:

(1) $$N_{D,W,Q_{\mathrm{cross}}}^{\mathrm{BPC}}=\frac{M_{Q_{\mathrm{cross}}}^{\mathrm{FC}}}{M_{Q_{\mathrm{cross}}}^{\mathrm{BPC}}}\times N_{D,w,Q_{0}}^{\mathrm{FC}}\times k_{Q_{\mathrm{cross}},Q_{0}}^{\mathrm{FC}}$$

$M_{Q_{cross}}^{BPC}$ and $M_{Q_{cross}}^{FC}$ represent the readings for BPC and FC, respectively, for the proton beam at quality $Q_{cross}$, corrected for temperature, air pressure, polarity effect, and ion recombination. $N_{D,w,Q_{0}}^{FC}$ represents the calibration factor of the FC for absorbed dose to water for a ^60^Co beam at quality $Q_{0}$, while $k_{Q_{\mathrm{cross}},Q_{0}}^{\mathrm{FC}}$ signifies the beam quality correction factor of the FC.

#### 2.3.2 Response of BPC to mono‐energetic beams

The long‐term response of the BPC to mono‐energetic beams was assessed (right side of Figure 1). Pencil beam measurements to the BPC are greatly influenced by spot position errors. To mitigate this effect, mono‐energetic beams were created as two‐dimensional fields. The measurement depth was 20 mm, with beam energies of 70.2, 144.1, and 228.7 MeV. Among these, 70.2 and 228.7 MeV represented the minimum and maximum energies of the proton treatment machine, respectively, while 144.1 MeV served as the intermediate energy between the minimum and maximum. The delivery beams had a field size of 11.4 × 11.4 cm^2^, spot spacing of 3 mm, MU per spot of 0.03, and a rescan number of three times. The BPCʼs responses were corrected for temperature, air pressure, polarity effect, and ion recombination.

#### 2.3.3 Correction factor of polarity and ion recombination effect

$k_{\mathrm{pol}}$ and *k_s_* were evaluated (left side of Figure 1) using only the same SOBP field (as in Section 2.3.1) because the polarity and ion recombination effect were found to be independent of linear energy transfer (LET) in proton beams.^16^ *k*_pol_ is defined as follows^6^:

(2) $$k_{pol}=\frac{\left|Q_{+}\right|+\left|Q_{-}\right|}{2\left|Q\right|}$$

|*Q*_+_| and |*Q*_–_| represent the charge collected at positive and negative polarity (+400 and –400 V, respectively). |*Q*| indicates the absolute charge collected for the regular polarity application (+400 V was used in this study). As the BPC charge collection time of 67 µs was significantly shorter than the spill time (up to 8 s), the beam delivery was considered continuous, and *k_s_* is defined as follows^6^, ^17^:

(3) $$k_{s}=\frac{{\left(V_{1}/V_{2}\right)}^{2}-1}{{\left(V_{1}/V_{2}\right)}^{2}-\left(M_{1}/M_{2}\right)}\;$$

*V*_1_ and *V*_2_ represent supplied voltages of +400 V and +200 V, respectively, and the readings at each applied voltage are *M*_1_ and *M*_2_, respectively.

#### 2.3.4 Impact of setup on response

To explore the influence of setup on the response of the chambers, dose measurements were conducted using both the FC and BPC with repeating the setup three times. The beam configuration and positioning of the chambers conformed to the procedures outlined in Section 2.3.1. On the same day, both the chambers and the water phantom were repositioned three times. For each setup configuration, dose measurements were performed threefold.

## 3 RESULTS

**FIGURE 2:**
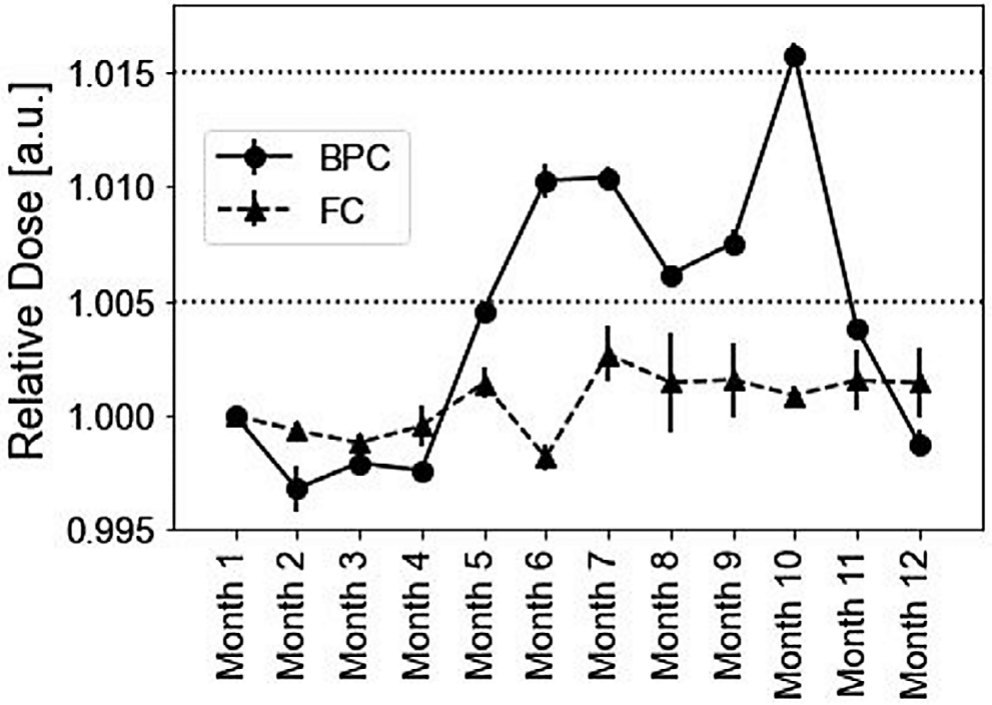
Temporal variation of the normalized FC and BPC response. Error bars indicate the standard deviation derived from the measurements, with results normalized relative to the values from Month 1.

**FIGURE 3:**
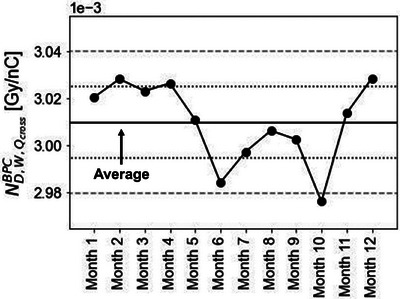
Temporal variation of $N_{D,W,Q_{\mathrm{cross}}}^{\mathrm{BPC}}$. The central solid line represents the average value of $N_{D,W,Q_{\mathrm{cross}}}^{\mathrm{BPC}}$. The outer and inner dash lines are at ±1.0% and ±0.5% relative to the mean.

Figure 2 shows the temporal variation in the BPC and FC over the course of one year, from December 2022 to November 2023, with the months labeled as Months 1–12. The error bars represent the standard deviation derived from the measurements. The results were normalized based on the values from Month 1. The variation observed in the FC results was 0.14% (1SD) and remained highly stable throughout the period. The discrepancy between the maximum and minimum measured values was 0.45%, consistent with the vendor specification of 0.5%. Furthermore, the absorbed dose to water calibration factor of the FC was calibrated in October of each year—2021, 2022, and 2023—by a dosimetry calibration laboratory in Japan. The calibration factors were 5.394E‐02, 5.387E‐02, and 5.392E‐02, respectively, and a negligible difference of 0.1% between the maximum and minimum values. In contrast, the variation in the BPC response was 0.61% (1SD), larger than of the FC. The discrepancy between the maximum and minimum measured values was 1.9%, and the variation over one month from Months 10 to 11 was 1.2%. However, the reproducibility of the measurements taken three times each month remained stable in both the FC and BPC, with maximum deviations of 0.2% and 0.1%, respectively. The temporal variation in $N_{D,W,Q_{\mathrm{cross}}}^{\mathrm{BPC}}$ is shown in Figure 3. The average value and variation of $N_{D,W,Q_{\mathrm{cross}}}^{\mathrm{BPC}}$ over the year were 3.01E‐3 and 0.57% (1SD), respectively. The difference between the maximum and minimum $N_{D,W,Q_{\mathrm{cross}}}^{\mathrm{BPC}}$ was 1.8%, exhibiting a monthly variation of 1.2% from Months 10 to 11.

**FIGURE 4:**
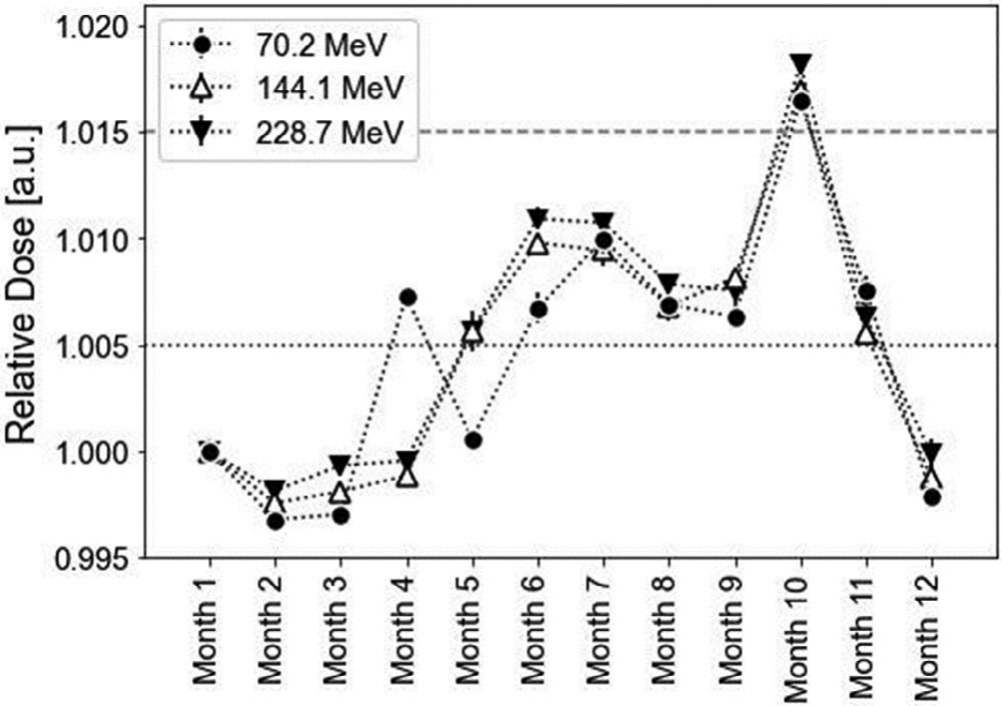
Temporal variation of relative dose of the BPC in the mono‐energetic beams. Error bars represent the standard deviations derived from the measurements. The doses were normalized to the values recorded in Month 1 for each energy level.

Figure 4 illustrates the temporal variation in the BPC response to the mono‐energetic beams. The doses were normalized to the measurement values of Month 1 for each energy measurement, with error bars representing the standard deviations of the measured values (The error bars are not visible due to overlapping with the symbols). The variation in measured values over time is 0.6% (1SD) for all energies. For the energy of 228.7 MeV, the difference between the maximum and minimum measured values was 2.0%, and the variation within one month from Months 10 to 11 was 1.2%. The reproducibility of the measurements, taken three times each month, remained stable, with a maximum deviation of 0.1% for all energies. The relative doses of all energies were within 1% each month, and the variation trend in the BPC was consistent across all energies. Furthermore, both the long‐term response and reproducibility of the BPC to both the SOBP field and mono‐energetic beams exhibited the same trend.

**FIGURE 5:**
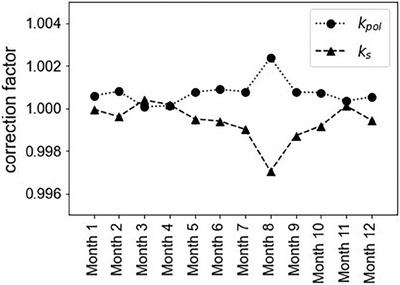
Temporal variations of *k*_pol_ and *k_s_* of the BPC.

Figure 5 shows the temporal variations in *k*_pol_ and *k_s_* of the BPC. The mean values of both *k*_pol_ and *k_s_* were 1.00 with 1SD of 0.09% and 0.06%, respectively. The annual variations in *k*_pol_ and *k_s_* were within 0.4% and remained relatively constant throughout the period.

Table 1 displays the responses of the FC and BPC across three repeated setups. The responses for both the FC and BPC have been normalized to their respective mean values. Across the three setups, the maximum variation in response was 0.2% for the FC and 0.1% for the BPC.

**TABLE 1:** Responses of the FC and BPC across three repeated setups.

	Setup		
Chamber	1st	2nd	3rd
FC (a.u.)	1.001	1.000	0.999
BPC (a.u.)	1.000	1.000	1.001

Figure S1 depicts the temperature of the water phantom and the atmospheric pressure in the treatment room throughout the measurement period. Throughout the year, the water temperature varied from 22.7°C to 25°C, while the atmospheric pressure fluctuated between 997.9 and 1020.9 hPa.

Table 2 demonstrates an uncertainty budget for $D_{W,Q}$, which is absorbed dose to water for the clinical proton beam quality Q. The table lists relative standard uncertainties at a confidence level of 68% (coverage factor *k* = 1). The type B uncertainties were referenced from TRS398.^6^ The BPC uncertainty includes that of cross‐calibration with the FC. The uncertainty of $D_{W,Q}$ for the BPC was larger than that for the FC.

**TABLE 2:** Estimated relative standard uncertainty o*f* $D_{W,Q}$ under a clinical condition.

	Type	FC	BPC
Step 1: standards laboratory			
$N_{D,w,Q_{0}}^{FC}$ calibration in a ^60^Co beam at a secondary standards dosimetry laboratory	B	0.6	
Step 2: cross‐calibration of BPC in clinical proton beam			
Ratio of dosimeter readings $M_{Q_{cross}}^{FC}/M_{Q_{cross}}^{BPC}$	A	―	0.2
Long‐term stability	A	―	0.61
Setup	A	―	0.06
Correction for influence quantities	B	―	0.3
Beam quality correction $k_{Q_{cross},Q_{0}}^{FC}$	B	―	1.6
Combined uncertainty in step 2		―	1.8
Step 3: clinical proton beam			
Reproducibility	A	0.2	0.1
Long‐term stability	A	0.14	0.61
Setup	A	0.1	0.06
Correction for influence quantities	B	0.3	
Beam quality correction $k_{Q,Q_{cross}}$	B	1.4	0.6
Combined uncertainty in step 3		1.5	1.0
Combined standard uncertainty in $D_{W,Q}$		1.6	2.1

$k_{Q,Q_{cross}}$: beam quality correction factor between Q and $Q_{cross}.$

## 4 DISCUSSION

The accuracy of the beam monitor in scanning beam delivery requires a tolerance of ±2% for high‐precision proton therapy. Achieving this tolerance underscores the significance of the long‐term stability of both the chamber and treatment machine. Despite the calibration of the absolute dose of PBS delivery using the BPC, the long‐term stability of the BPC to clinical proton beams has remained uncertain. Hence, this study aimed to evaluate the long‐term stability of the BPC using clinical proton beams over the course of a year.

The annual variation observed in the reference chamber (referred to as the FC) within the SOBP field remained within 0.5%. This aligns with the annual validation of 0.5% for the Strontimu‐90 source,^18^ indicating the reliability of the FC results obtained in this study. However, the variations observed in the FC in this study were influenced by the fluctuations in the proton beam output. Even if the variation in the proton beam output was potentially overestimated, it was assumed to be less than 0.5% because the variation in the FC remained within this range. Furthermore, spot sizes of the proton beam were assessed monthly using the cintillation detector (Logos Systems Int'l, USA) as shown in Figure S2. The deviation from reference data was 3%, which falls within the acceptable guideline tolerance.^11^ Therefore, it can be concluded that the output of the clinical scanning beams was sufficiently accurate.

Responses of the FC and BPC from three repeated setups revealed maximum variations of 0.2% and 0.1%, respectively. These figures align with the repeatability observed in the monthly cross‐calibration measurements conducted for both chambers, indicating minimal variations in the measurements for both chambers. Consequently, the influence of setup on the responses of the FC and BPC is considered to be negligible.

The annual variation observed in the cross‐calibration factor and responses of the SOBP field and mono‐energetic beams were 1.8%, 1.9%, and 2.0%, respectively. Table 3 presents a comparison of the long‐term stability between our study and existing reports.^18^, ^19^ As depicted in Table 3, the long‐term stability of the BPC was found to be inferior to that of the FC in our study and previous reports. Conversely, the long‐term variations in the BPC for the SOBP field and mono‐energetic beams were consistent (Figures 2 and 4). This observation implies that the response of the BPC varies similarly for different beam energies and measurement depths, even under limited conditions. Therefore, it can be inferred that the response of the BPC could be independent of the clinical proton beam. In addition, the reproducibility of the BPC was found to be very high, with a maximum deviation of 0.1% observed for both the SOBP field and mono‐energetic beams. In summary, despite its inferior long‐term stability compared to the FC, the BPC can still be reliably used to measure relative doses due to its high reproducibility.

**TABLE 3:** Comparison of the long‐term stability of the chambers.

Chamber	BPC	BPC	FC	FC^18^	PPC^19^
Beam	SOBP	Mono energy	SOBP	^90^Sr	^90^Sr
Long‐term stability (%)	1.9	2.0	0.45	0.39	0.5

Abbreviation: PPC, PinPoint chamber.

The long‐term variations of *k*_pol_ and *k_s_* remained stable (within 0.4%) throughout the investigation period. These correction factors maintain stability as long as the beam characteristics remain unchanged. Considering that *k*_pol_ and *k_s_* are not dependent on LET and energy,^16^ their long‐term variations are minimal at any depth, consequently exerting a small impact on the response of the BPC. Thus, the effects of polarity and ion recombination on the long‐term stability of the BPC were negligible.

Given the findings of this study suggesting that the impact of clinical proton beams and setup on the BPC response is minimal, it is likely that the variations observed are primarily attributable to the BPC itself. The BPC is one of the parallel‐plate chambers, but previous research indicates that the long‐term stability of parallel‐plate chambers is inferior to that of FCs.^18^, ^20^, ^21^, ^22^ This inferior stability can be attributed to factors such as the chamber structure^23^ and environmental conditions.^24^, ^25^ Therefore, it is plausible that the significant variation in the BPC's response may stem from similar sources. In particular, the BPC may exhibit heightened sensitivity to environmental conditions owing to its larger volume compared to other parallel‐plate chambers. Moreover, the response of a parallel‐plate chamber is highly susceptible to changes in the distance between the electrodes. For instance, even a minimum alteration of 20 µm in the distance between the plates can lead to a 1% change in response.^26^ Given the sensitivity to such micro‐scale changes, variations in the BPC's response may be induced by alterations in the volume of the chamber cavity of the chamber cavity or the distance between the plates arising from environmental conditions.

To assess the accuracy of the beam monitor in PBS delivery, it is important to consider not only the delivery machine but also the long‐term stability of the chambers. Another critical factor is ensuring minimal energy dependency of the chambers, given the wide energy range utilized in proton therapy. In this study, variations in energy dependence, polarity effect, and ion recombination effect were found to be negligible within the clinical energy range. However, the observed annual variation in the BPC's response, reaching up to 2.0%, is deemed unacceptable, considering the tolerance of the QA guidelines. Neglecting the long‐term stability of the BPC during the TPS dose calibration could introduce a total error of 2.0% in delivered doses. Despite the BPC's demonstrated high reproducibility over an extended period, with a standard deviation within 0.1% for each measurement, its consistency with that of the FC suggests its suitability for long‐term relative dosimetry. Consequently, while the BPC can serve for absolute doses measurements, it is essential to cross‐calibrate it with the reference chamber for each measurement. Even if the BPC is calibrated in a dosimetry calibration laboratory, its response should be systematically compared with that of the reference chamber, considering any variations in the BPC's response. This approach mirrors the recommended practice for absolute dosimetry of photon and electron beams using parallel‐plate chambers.^25^ Therefore, it is crucial to correct the BPC's response to match that of the reference chamber in each measurement to ensure accurate absolute dose measurements.

The absolute dose uncertainty of the BPC was larger than that of the FC (Table 2). This is due to the uncertainties of the BPC in cross‐calibration and long‐term stability. Therefore, when using the BPC for QA procedure in absolute dose, this uncertainty should be considered in dose evaluation.

TRS398^6^ recommends performing cross‐calibration using the highest energy in the plateau region. However, this study employed FC as the reference chamber and due to its width in the depth direction, cross‐calibration was conducted in the flatter center of the SOBP rather than in the plateau region. Moreover, the maximum energy of the SOBP field in this study was 218.7 MeV, which is lower than the maximum available energy of 228.7 MeV in the treatment machine. Although there is a discrepancy of 10 MeV from the TRS398 recommendation, the response of the BPC at the mono‐energetic beam of 228.7 MeV was similar to its response in the SOBP field. Therefore, despite the deviation from the TRS398 guidelines, the results of this study are considered reliable.

In this study, a single BPC was utilized to investigate the long‐term stability using clinical proton beams. Another study^13^ demonstrated that the long‐term stability of the BPC was within 0.6% over 9.5 months, employing a Strontimu‐90 source. Although the types of beams differed between the studies, variations in variability could potentially be attributed to individual differences among BPCs. Thus, other BPCs may exhibit greater variations compared to the one examined in this study. Furthermore, the response within the BPC is inhomogeneous^1^, ^26^, ^27^; however, this study did not include inhomogeneity corrections within the BPC. Therefore, with such corrections, the variation in BPC response could be potentially larger. The specific variation within different regions of the BPC volume remains unclear. Evaluating the long‐term stability within each region of the BPC volume could unveil its detailed characteristic. Implementing inhomogeneity corrections within the BPC for long‐term stability may enhance the accuracy of measuring the absolute dose of proton scanning beams.

## 5 CONCLUSION

In this study, the long‐term stability of the BPC was evaluated using clinical proton scanning beams over a span of one year. Notable variations were observed in the response and cross‐calibration factor of the BPC, with maximum changes of reaching 1.9% and 1.8%, respectively. In contrast, the response of the BPC remained unaffected by the delivery beam and measurement depth, and minimal variations were noted in the polarity and ion recombination effects. These findings affirm that the BPC is suitable for relative dose measurements over long periods. Consequently, when employing the BPC for absolute doses measurements, it is imperative to compare and correct its response to that of the reference chamber for each measurement.

## AUTHOR CONTRIBUTIONS

Masashi Yamanaka and Yutaro Mori made substantial contributions to the conception of the study. Kazuki Matsumoto, Shunsuke Moriya, Akihiro Yamano, Takahiro Shimo, Ryosuke Shirata, Kazunori Nitta, Hironori Nagata, and Koichi Tokuuye made significant contributions to the data analysis and interpretation. Masashi Yamanaka and Yutaro Mori drafted the original manuscript. All authors critically reviewed and revised the manuscript draft and approved the final version for submission.

## CONFLICT OF INTEREST STATEMENT

The authors declare no conflicts of interest.

## Supporting information



FIGURE S1 Temporal variation of the water phantom temperature and atmospheric pressure in the treatment room.

FIGURE S2 The spot size was assessed monthly using the XRV‐2000 scintillation detector over the entire period. Each month, a single spot beam was irradiated at energies of 70.2, 114.1, and 228.7 MeV. The average spot sizes measured on the X‐axis and Y‐axis were 7.04 mm and 7.16 mm at 70.2 MeV, 3.52 mm and 3.65 mm at 114.1 MeV, and 2.50 mm and 2.56 mm at 228.7 MeV, respectively. Figure S2 illustrates the discrepancies in spot size relative to the reference data, which remained within a 3% error margin for all energies.
