# Supplementary material for: Long‐term stability of a PTW 34070 large‐area parallel ionization chamber in clinical proton scanning beams
Source: J Appl Clin Med Phys. 2024 Sep 16;25(12):e14525. doi: 10.1002/acm2.14525 (PMC11633811; doi:10.1002/acm2.14525)
Supplement: Supplementary file 1 — FIGURE S1 Temporal variation of the water phantom temperature and atmospheric pressure in the treatment room. [file ACM2-25-e14525-s002.pptx]

## Slide 1
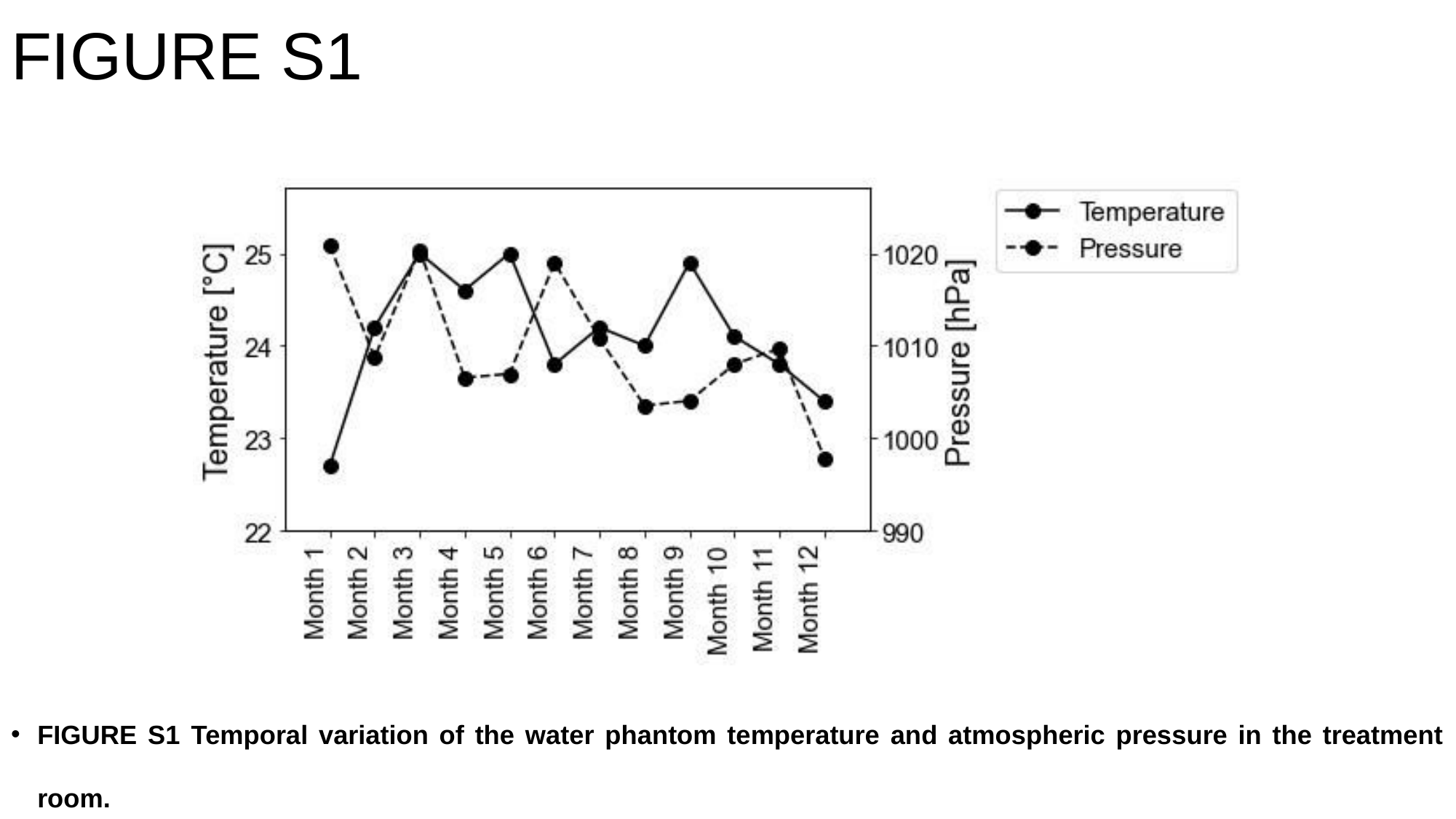

# FIGURE S1
FIGURE S1 Temporal variation of the water phantom temperature and atmospheric pressure in the treatment room.
