# Supplementary figures and images for: Long‐term stability of a PTW 34070 large‐area parallel ionization chamber in clinical proton scanning beams
Source: J Appl Clin Med Phys. 2024 Sep 16;25(12):e14525. doi: 10.1002/acm2.14525 (PMC11633811; doi:10.1002/acm2.14525)

## Slide 1
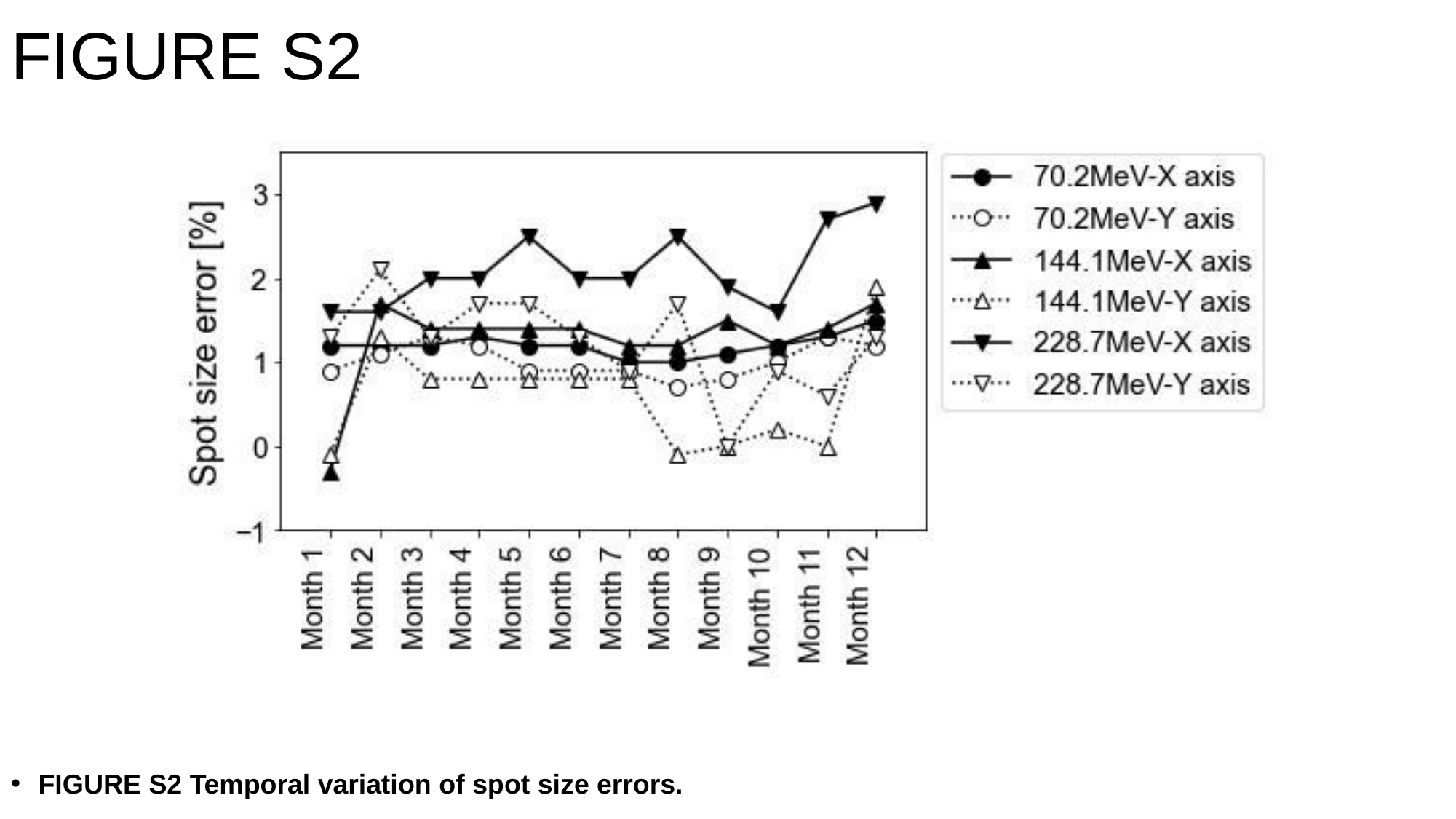

# FIGURE S2
FIGURE S2 Temporal variation of spot size errors.

Supplement: Supplementary file 2 — FIGURE S2 The spot size was assessed monthly using the XRV‐2000 scintillation detector over the entire period. Each month, a single spot beam was irradiated at energies of 70.2, 114.1, and 228.7 MeV. The average spot sizes measured on the X‐axis and Y‐axis were 7.04 mm and 7.16 mm at 70.2 MeV, 3.52 mm and 3.65 mm at 114.1 MeV, and 2.50 mm and 2.56 mm at 228.7 MeV, respectively. Figure S2 illustrates the discrepancies in spot size relative to the reference data, which remained within a 3% error margin for all energies. [file ACM2-25-e14525-s001.pptx]
